# From village health volunteers to paid care givers: the optimal mix for a multidisciplinary home health care workforce in rural Thailand

**DOI:** 10.1186/s12960-020-00542-3

**Published:** 2021-01-06

**Authors:** Nonglak Pagaiya, Thinakorn Noree, Penapa Hongthong, Karnwarin Gongkulawat, Pagaluk Padungson, Dariwan Setheetham

**Affiliations:** 1 Faculty of Public Health, Khon Kaen University, 123 Mitraphap road, Muang, 40002 Khon Kaen Thailand; 2 International Health Policy Program, Ministry of Public Health, Tiwanon road, Muang, 11000 Nonthaburi Thailand

**Keywords:** Home health care, Elderly, Care giver, Multidisciplinary health team

## Abstract

**Background:**

Thailand is a rapidly aging society, which places high demand on home health care services for the elderly. The shortage of health care workforce in rural areas is a crucial obstacle to the delivery of adequate home health care services. The appropriate skill-mix between multidisciplinary health team and care givers (CGs) is an attractive solution for improving home health care services in rural Thailand. This study assessed the potential of trained CGs to provide home health care services and projected what the optimal mix for a multidisciplinary home health care team in rural Thailand would be in 2030.

**Methods:**

Eleven pilot districts in Thailand were recruited for the study. Secondary data were collected along with surveys of home health care providers. A total of 130 care managers (nurses) and 351 care givers (CG) were recruited for the survey. Workload, skill-mix potential, and acceptance of care givers were assessed in the surveys. The results from secondary data and the survey were used to project the health workforce requirements in 2030.

**Results:**

It is projected that in 2030 the number of elderly living in rural areas will be 7,156,700 (27% of the projected rural population). Of this, 20.3% will be home-bound, 1.1% will be bed-ridden and 1.6% will need rehabilitation. The main members of the multidisciplinary health workforce involved in home health care were nurses, doctors, and physiotherapists. The home health care services that were provided by the multidisciplinary health workforce included patient assessment, development of a care plan and case conference, home visits, and teaching and supervision of CGs. The CGs were village health volunteers trained to carry out regular home visits to patients. The CGs provided assistance with the activities of daily living, basic health services, moral support to patients and relatives, and surveillance of the home environment during home visits. CGs were well accepted by both the health professionals and the patients. Projections showed that 16,094 nurses, 1,542 doctors, 1,022 physiotherapists and 50,148 CGs will be required in 2030 to meet the needs of the dependent elderly for home health care in rural Thailand.

**Conclusion:**

With the increased need for home health care services in the future, appropriate team work between the members of the multidisciplinary health team and the CGs in the community is the appropriate solution for likely shortages of health professional workforce.

## Background

A transition in socio-demographics is leading to growing proportions of elderly in the world’s population. Accompanying this are rising numbers of chronic diseases, multiple-morbidities and age-related immobility meaning long-term care (LTC) needs have been increasing in both developing and developed countries [1–3]. LTC encompasses the range of services required by dependent persons for an extended period of time. LTC includes assistance with the activities of daily living (such as bathing, dressing, and eating) in combination with basic medical services (such as wound dressing, pain management, medication, health monitoring, prevention, rehabilitation, and palliative care). LTC services also include care related to the instrumental activities of daily living (IADL), such as help with housework, meals, shopping, and transportation [2]. As the number of elderly and the range of services that need to be provided in LTC has increased, health costs have also increased and many governments are now encouraging older people to live at home for as long as possible, supported by their social network and home care [1, 2, 4, 5].

In Thailand, the number of older persons (defined as aged 60 and over) has grown from approximately 8.4 million in 2010 to 12.6 million in 2020 or 19.1% of the total population (66 million). Future population aging will occur even more rapidly with the number of older persons projected to increase to nearly 17.6 million by 2030, at which point they will constitute 26.6% of the Thai population [6, 7]. Although most elderly people live independently, 21.4% are considered dependent on LTC services according to the Barthel Activities of Daily Living (ADL) Index [8]. This suggests that there will be a dramatic increase in the need for LTC in the future.

Home care for elderly people is mostly provided informally by life partners and adult children, but social changes are weakening this social support network. Increased mobility has led to a wider geographical separation between parents and children, particularly for parents in rural areas. These socio-economic changes have been accompanied by a continuous reduction in fertility rates and a greater number of women entering labor market. The Thai National Statistic Office conducted a survey in 2017 that showed 11% of elderly lived alone and 22% lived with only their life partner [6]. To encourage and support the elderly to stay at home, the Thai government implemented a policy in 2016 to deliver LTC services to independent elderly in the home [9]. With this policy, home-bound and bed-ridden elderly can receive regular home health care services by paid care givers and multidisciplinary health teams. Paid care givers (CG) are trained village health volunteers who provide support services, personal assistance and health services to elderly living in their community with technical support from a multidisciplinary health team consisting of doctors, nurses, and physiotherapists. Paid care givers (CG) are non-professionals who are recruited from communities and trained by the multidisciplinary team in a 70-h CG training course [9]. Since rural areas have a unique set of challenges concerning elderly care as well as with the health service system, this study has focused on the home health care system in rural areas.

The rural health service facilities that deliver services to people comprise health centers and rural hospitals. Health centers are the first contact with the health care system for individuals, families and communities. The services provided by health centers incorporate common illness care, health promotion, disease prevention, rehabilitation, and community health interventions. LTC services for the dependent elderly are included as primary care services provided by health centers. Rural hospitals work closely with health centers to deliver care to rural people, and to provide technical support to health centers and secondary care services [10]. The services at health centers are mainly provided by nurses, public health officers and dental nurses. Doctors, dentists, pharmacists, physiotherapists, and medical technologists are based at rural hospitals but render support services regularly as needed. In communities and villages, village health volunteers (VHV) are available to look after 10 households each.

To provide effective home health care, a multidisciplinary team of key health professionals assesses patients, plans for care provision, carries out home visits, supervises CG and monitors patient situations. However, the shortage of health care providers, particularly in rural areas, is a crucial obstacle to the success of the home health care system. In terms of health workforce numbers, it is projected that in 2026, the overall number of health care providers will meet requirements in most professions in Thailand, but there will be a shortage of around 47,000 nurses [10]. However, the health workforce challenges are not limited to shortages in supply, but also inequitable distribution and high turnover. Although 54% of the Thai population reside in rural areas, only 16.5% of doctors work there as a high proportion of doctors leave rural areas [11]. These problems are repeated for physiotherapists (PT) with only 18.2% of PT working in rural areas and about one-fourth intending to leave public facilities [12].

Paid care givers are an attractive proposition for providing accessible and qualified services to dependent elderly at home. In such a limited health workforce, the optimal skill-mix between the main stream health professionals and the paid care givers needs to be defined. Therefore, we assessed the potential of paid care givers to provide home care services and projected the personnel requirements for a multidisciplinary health workforce incorporating paid care givers to deliver effective elderly home health care in rural Thailand in 2030.

## Methods

This descriptive study used the primary data together with the secondary data in order to determine the current situation of home health care services and the health workforce and then projected the health workforce requirements for home health care services in rural Thailand. As the Thai government policy emphasizes support for dependent elderly in their homes and preventing or delaying any loss of function [9], the key health care professionals included in this study were nurses, doctors and physiotherapists (PT). In addition, CG were included as the key workforce at the village level. The process of data collection and analysis followed two steps.

### Situation analysis

One district from each of the 11 health areas in Thailand was recruited as the study sites. Inclusion criteria included (1) being an LTC-piloted district since 2016, (2) volunteer to participate in the study. Therefore, 11 districts were included. All of the care managers (CM, nurses) and a sample of the CG from each district were included in the survey. In total, 130 care managers from 11 districts were recruited for data collection. From a total of 841 CG, the sample size calculation at the 95% confidence level showed that 351 CG were required. To achieve a total of 351 CG, the proportion of CG recruited from each district was 41.7% (351/841) of the CG in a district, i.e., 33 CG would be recruited from a district with a total of 80 CG. A self-administered questionnaire for CM was developed to assess LTC services and workload, skill-mix potential, acceptance of CG and service outcomes. The self-administered questionnaire for CG comprised general information, services carried out, opinion towards working as CG and intention to stay.

Service outcomes were measured in relation to patient outcomes, CG acceptance and CG turnover rate.

With regards to patient outcome measurement, the Thai Ministry of Public Health (MoPH) has adapted the Barthel Activity of Daily Living (ADL) index [13] to assess patient function based on 10 activities: self-feeding (0–2 points), grooming (0–1 point), transfer (0–2 points), toilet hygiene (0–3 points), functional mobility (0–3 points), dressing (0–2 points), stairs (0–2 points), bathing (0–1 point), control of bowels (0–2 points) and bladder (0–2 points). Zero points means the patient cannot perform the function by themselves, higher points indicate a greater likelihood of being able to live at home with a degree of independence. Following assessment, each patient was categorized as either bed-ridden (0–4 points), home-bound (5–11 points) or independent (12 points and above).

CG acceptance was assessed by self-administered questionnaires to CM and CG. CG turnover was assessed from the CM questionnaire and CG intention to stay was assessed by the CG questionnaire.

Researchers contacted the head CM of each district to obtain the name codes and addresses of all CM and CG. The self-administered questionnaires were then mailed directly to the participating CM and CG and they were returned directly to the researchers after completion. The questionnaire collection period took three months.

### Health workforce requirements

The results from step 1 were used together with secondary data to project the health workforce requirements for home health care services in 2030 according to the following steps.

1. Assess the projected number of rural dependent elderly in relation to elderly population size and dependence level in 2030. The projection for the number of dependent elderly was based on the National Health Statistics Survey Report [6, 7] and the National Health Examination Survey [8]. Details are shown in the results section.
2. Identify the services provided and the time spent by each professional based on the survey results from step 1. Since the services that are needed by dependent elderly involve integrated care, a multidisciplinary team is required and the skill-mix between the health professionals and CGs needs to be considered. The key health care workforce included in this study were nurses, doctors, physiotherapists (PT) and CG. During the analysis process, a range of services was identified for each type of provider (nurse, doctor, PT or CG). The time spent by each care provider for each service was identified from the information gathered in step 1.
3. Calculate the annual working hours for each cadre based on the number of dependent elderly people in 2030, the volume of each activity carried out, and the time spent with each activity.
4. The health workforce requirement was determined by using the staffing standard for 1 full time equivalent (FTE) per year (1680 h) [14]. The annual working hour calculation consisted of 7 h per day for 240 days per year. Vacations and periods of leave were excluded.

## Results

Of 130 CM questionnaires, 107 were returned, which accounted for an 82.3% response rate. Each CM was responsible for LTC services of each sub-district. Overall, for 11 study districts, there were 88,291 elderly. Dependent elderly comprised 2,563 home-bound elderly and 632 bed-ridden elderly. There were 24.7 home-bound elderly and 6.1 bed-ridden elderly per sub-district. In relation to the health workforce, there were 112 nurses who also worked as CM, 11 doctors and 11 PT. In addition, there were 841 CG working at the villages. The ratios of nurses, doctors, PTs and CGs to dependent elderly were 1:28.3, 1:288, 1:288 and 1:3.8, respectively. The tasks of nurses, doctors and PT included some other health care services beside LTC services. See Table 1.

**Table 1:** Number of health workforce and ratio to the elderly

Health workforce	Number	Ratio
CM (nurses)	112	1:28.3
Doctors	11	1:288
PT	11	1:288
CG	841	1:3.8

### Service provision

The survey showed that main health care professionals involved with home health care were nurses (as CM), doctors and physio-therapists (PT). The main activities that they performed were patient assessment, case conference, home visit and CG supervision. For home-bound elderly, nurses assessed the patients, developed care plans, conducted case conferences, and trained and supervised CGs. Doctors were involved in case conferences. For bed-ridden cases, nurses carried out the same activities, but at higher frequencies. Doctors, in addition to providing support on case conferences, were also involved in home visits with bed-ridden patients. With regards to elderly who needed rehabilitation, PTs assessed their needs, developed care plans, carried out home visits, which included teaching and supervising CGs. CGs provided home visit services according to the plan of the multidisciplinary team. In addition to the training that they received to become CGs, CGs were taught on a case-by-case basis by members of the multidisciplinary team during home visits. Home-bound elderly were assessed every 3 months and received a home visit from nurses once a month. Home visits for bed-ridden elderly were once a month by doctors and twice a month by nurses, and case assessments were made monthly. CG home visits were weekly for home-bound patients and twice a week for bed-ridden patients. For rehabilitation services, patients received intensive services during a 3-month period. PTs made a monthly plan, carried out home visits and supervised CGs weekly, and CGs provided rehabilitation services over 4–5 visits per week. Time spent for each activity was the average time derived from the survey and rounded up into simple number. The frequency of each activity of each cadre, as well as the time spent is shown in Table 2.

**Table 2:** Services and time spent per year by multidisciplinary health teams by type of dependent elderly

Services/activities	Home-bound elderly quantity (h)			Bed-ridden elderly quantity (h)			Rehabilitation quantity (h)	
	Nurse	Doctor	CG	Nurse	Doctor	CG	PT	CG
Patient assessment	4 (0.5)			12 (0.5)			3 (0.5)	
Care plan	4 (0.5)			12 (0.5)			3 (0.5)	
Case conference	4 (0.25)	4 (0.25)		12 (0.25)	12 (0.25)			
Home visit^a^	12 (1.0)	–	52 (1.0)	24 (1.0)	12 (1.0)	104 (1.0)	12 (1.0)	24 (1.0)
CG supervision^a^	12		52	24	12	24	12	12

^a^ Home visit and CG supervision were carried out at the same time

### CG services and acceptance

CG received initial training to prepare them to assist dependent elderly at home for 70 h according to the CG training course developed by the Thai Ministry of Public Health (MoPH) [15]. The training cost was covered by the MoPH. Subject areas covered in training were personal care, disease-specific care, medications, nutrition, mental health, health promotion, and basic rehabilitation. Training incorporates 50 h of classroom lectures and 20 h practicum. On-the-job training was also provided by multidisciplinary team members during the home visits.

Of the 351 CG questionnaires sent, 304 were returned making an 86.6% response rate. The majority of CGs were female (90.1%). Few of them were young (aged 20–29 years; 4.3%), most of them were aged 40–59 (68%) and 11.6% were elderly (60 years and above). Career-wise, slightly more than half of them were farmers followed by odd jobs (19.0%) and housewife (17.6%), respectively. In terms of education, the majority of the CGs had completed secondary school (55.1%), followed by primary school (29.0%), with some completing vocational school or bachelor degree or higher. Village health volunteers (VHV) made up 91.8% of the CGs, and they were currently still working as VHV. CGs were paid based on the number of dependent elderly people in their charge. On average, they were paid around 1.7- 3.3 USD per patient visit. Almost half of them received around 15–30 USD per month, and 45% of them received 31–60 USD per month. Details are in Table 3.

**Table 3:** CG characteristics

CG characteristic	Number (%)
Gender	
Male	30/303 (9.9%)
Female	273/303 (90.1%)
Age	
20–29 years	13/303 (4.3%)
30–39 years	49/303 (16.1%)
40–49 years	120/303 (39.6%)
50–59 years	86/303 (28.4%)
60 years and above	35/303 (11.6%)
Career	
Farmer	156/301 (51.8%)
Odd jobs	57/301 (19.0%)
Housewife	53/301 (17.6%)
Others	35/301 (11.6%)
Education	
Primary school	88/303 (29.0%)
Secondary school	167/303 (55.1%)
Vocational school	22/303 (7.3%)
Bachelor’s degree and higher	26/303 (7.6%)
Work experience before becoming CG	
VHV	279/304 (91.8%)
Elderly assistant volunteer	8/304 (2.6%)
Others	17/304 (5.6%)
CG monthly wage	
15–30 USD	149/304 (49.0%)
31–60 USD	137/304 (45.1%)
61–90 USD	4/304 (1.3%)
91–120 USD	8/304 (2.6%)
121 USD and above	6/304 (2.0%)

The services provided by CGs covered a comprehensive mix of the needs of dependent elderly including: health services, social support, activities of daily living, and housing and environment surveillance. In home visit services, most of the activities carried out were assisting with the activities of daily living, providing moral support to patients and their families and advising on health promotion and prevention. Basic rehabilitation services were also provided according to the PT plan and wound dressing was provided to some specific case according to the care plan. Details are in Table 4.

**Table 4:** Services provided to dependent elderly by CG

Aspects	Service provision	Number (%)
Activities of daily living	Assist with activities of daily living	250/304 (82.2%)
Social	Provide moral support to patients and care givers	243/304 (79.9%)
Health services	Advise on health promotion and prevention	228/304 (75.0%)
	Basic rehabilitation services	161/304 (53.0%)
	Wound dressing/medication advice	24/304 (7.9%)
Environment	Housing and environmental surveillance	58/304 (19.1%)

### CG performance

The CGs and CMs opinions about the CG’s performance were generally in agreement (Table 5). Both CMs and CGs perceived that the provision of home health care in the communities by CGs was beneficial to the health system through increased accessibility to services and reduced health workforce workloads. The majority of CMs and CGs reported that they believed CGs were well accepted by patients, had a good attitude towards patients and received adequate support from CMs. More than half of the respondents agreed that recruitment of CGs was properly conducted as CGs were principally recruited from the VHV. Slightly less than half of the CMs and CGs responded that they believed CG’s skills were sufficient. There were also some differences in the CGs and CMs opinions about the CG’s performance. Half of the CM respondents considered there were enough CGs compared to the workload, but only one third of CGs believed this. CMs and CGs also differed in their responses to CG training and retention. The majority of CGs believed that CGs received adequate training, whilst less than half of the CMs thought CG training was sufficient. More than half of the CGs reported that they thought CGs could be retained to work for LTC in the long term, but only one quarter of CMs believed this. Finally, CGs and CMs were in agreement concerning CG wages, with only one quarter of respondents agreeing that CGs obtained an adequate wage (Table 5).

**Table 5:** Opinions of CM and CG towards selected aspects of CG performance

CG performance	Agreed responses	
	CM, *n*/*N* (%)	CG, *n*/*N* (%)
Having CG increased accessibility to LTC services	76/96 (72.4)	237/300 (79.0)
Having CG reduced health workforce workloads	73/96 (69.5)	212/292 (72.6)
CG was accepted by the patients and their relatives	69/96 (65.7)	227/298 (76.2)
CG had good attitude towards the patients	71/95 (67.0)	219/300 (73.0)
CG received adequate support from CM	69/96 (66.3)	213/292 (72.9)
CG recruitment was properly conducted	60/96 (57.1)	173/298 (58.1)
Number of CG was adequate for workload	55/96 (51.9)	98/302 (32.5)
CG received adequate training	47/95 (44.3)	198/300 (65.3)
CG had sufficient skills	46/95 (43.4)	144/300 (48.0)
CG was properly retained to work for LTC in the long term	30/96 (28.6)	196/295 (66.4)
CG’s wage was adequate	25/96 (27.5)	79/285 (27.7)

### Service outcomes

In 2016, 955 people underwent training to become CG, and 114 (11.9%) of them had left the profession by the end of 2018. In our survey, approximately 16% of the current CGs intended to leave CG work in the next 2 years. The reasons given for those intending to leave were; being too old to continue working and not in good health, looking for a higher paid job, and being too busy with their main work. In terms of patient outcomes after receiving home health care services from multidisciplinary teams and CGs, the ADL scores at the end of 2018 were compared to scores at the beginning of 2018. The majority of the elderly patients in the survey maintained their ADL score at the end of the year, with 14.3% showing improved ADL scores and 10.1% having lower ADL scores over the twelve months (Table 6).

**Table 6:** CG turnover and elderly outcomes in 2018

Service outcomes	Number	%
CG turnover during 2018	114/955	11.9
CG intention to leave next 2 years	49/297	16.1
Elderly ADL changes		
All dependable elderly	3195	
Maintain the same ADL level	2417	75.6
ADL level improved	457	14.3
ADL level decreased	321	10.1

### Projected home health care workforce requirements for rural elderly

To determine the requirements of the home health care workforce team (nurses, doctors, PT and CG) in 2020 and 2030, secondary data [6–8, 16] and primary data from the survey were used. The National Statistical Office [6] projects that there will be 6,337,600 and 7,156,700 elderly people residing in rural Thailand in 2020 and 2030, respectively. The proportions of home-bound elderly, bed-ridden elderly and elderly with rehabilitation needs were derived from the 2014 National Health Examination Survey [8] and the 2013 MoPH health survey report into Thai elderly health [16]. The surveys reveal that the proportions of the elderly population who are home-bound and bed-ridden amounted to 20.3% and 1.1% of the elderly population, respectively. To estimate the proportion of elderly people with rehabilitation needs, we used the proportion of the elderly population who have suffered a stroke [8] as a proxy. Details are shown in Table 7. The activities and services carried out in home health care visits and the time spent per activity by each professional are indicated in Table 2.

**Table 7:** Baseline information for health workforce projection in rural areas, Thailand

Type of data	Number/%
Dependent elderly	
Home-bound elderly	20.3% of elderly population
Bed-ridden elderly	1.1% of elderly population
Elderly with rehabilitation needs	1.6% of elderly population
Projected elderly in 2020	
All elderly	6,337,600
Home-bound elderly	1,248,507
Bed-ridden elderly	69,714
Rehabilitation needs	101,402
Projected elderly in 2030	
All elderly	7,156,700
Home-bound elderly	1,409,870
Bed-ridden elderly	78,724
Rehabilitation needs	114,507

In 2020, it is projected that there will be 1,248,507 home-bound elderly and the key members of the multidisciplinary health teams delivering services are nurses, doctors, and CGs. From our survey, patient assessment by nurses took 0.5 h per case for a total of 2,497,014 working hours per year, with four assessments per year. Nurses also developed care plans, managed case conferences, and conducted home visits, which equaled 249,014 working hours, 1,248,507 working hours and 14,982,086 working hours per year, respectively. The total working hours was converted into the number of nurses required to provide these services by dividing by the staffing standard hours of 1 full time equivalent (FTE) nurse per year or 1,680 working hours. The nursing requirement was 12,633.7 FTE nurses. For doctors, their case conference hours for home-bound patients totaled 1,248,507 working hours, which means 743.2 FTE doctors would be required. The total number of CG working hours for home visits was high (64,922,374 working hours), and the calculation showed that 38,644.3 FTE CGs would be required to provide these services. For the 69,714 bed-ridden elderly in 2020, the workloads for nurses, doctors and CGs were 2,718,830, 1,045,704, and 7,250,214 working hours equating to 1 618.4 FTE nurses, 622.4 FTE doctors, and 4 315.6 FTE CGs, respectively. Finally, 905.4 FTE PT and 1,448.6 FTE CG would be required to provide rehabilitation services for 101,402 elderly needing rehabilitation in 2020. See Table 8.

**Table 8:** The projected workload and health workforce requirements to provide LTC for the dependent elderly in 2020

Services/activities	Home-bound elderly			Bed-ridden elderly			Rehabilitation needs	
	Nurse	Doctor	CG	Nurse	Doctor	CG	PT	CG
Patient assessment	2,497,014			418,282			152,102	
Care plan	2,497,014			418,282			152,102	
Case conference	1,248,507	1,248,507		209,141	209,141			
Home visit and CG supervision	14,982,086	-	64,922,374	1,673,126	836,563	7,250,214	1,216,819	2,433,638
Total	21,224,622	1,248,507	64,922,374	2,718,830	1,045,704	7,250,214	1,521,024	2,433,638
Health workforce requirement (FTE)	12,633.7	743.2	38,644.3	1618.4	622.4	4315.6	905.4	1448.6

The calculation of the FTE requirements for each profession to provide home health care to the dependent elderly in 2030 followed the same approach as above. The provision of LTC services for the projected 1,409,870 home-bound elderly in 2030 will require 14,266.5 FTE nurses, 839.2 FTE doctors and 43,638.8 FTE CG. The 78,724 bed-ridden elderly will require 1827.5 FTE nurses, 702.9 FTE doctors and 4873.4 FTE CGs. In addition, 1022.4 FTE PT and 1635.8 FTE CG will be required to look after the 114,507 elderly people needing rehabilitation. Details are shown in Table 9.

**Table 9:** The projected workload and health workforce requirements to provide LTC for the dependent elderly in 2030

Services/activities	Home-bound elderly			Bed-ridden elderly			Rehabilitation needs	
	Nurse	Doctor	CG	Nurse	Doctor	CG	PT	CG
Patient assessment	2,819,740			472,342			171,761	
Care plan	2,819,740			472,342			171,761	
Case conference	1,409,870	1,409,870		236,171	236,171			
Home visit and CG supervision	16,918,439	-	73,313,235	1,889,369	944,684	8,187,265	1,374,086	2,748,173
Total	23,967,789	1,409,870	73,313,235	3,070,224	1,180,856	8,187,265	1,717,608	2,748,173
Health workforce requirement (FTE)	14,266.5	839.2	43,638.8	1827.5	702.9	4873.4	1022.4	1635.8

Overall, in 2020, the LTC system will need 14,252 FTE nurses, 1366 FTE doctors, 905 FTE PT and 44,952 FTE CG. In 2030, to meet the needs of the dependent rural elderly, the LTC system will require a total of 16,094 nurses, 1542 doctors, 1022 PT and 50,148 CG as indicated in Table 10.

**Table 10:** Projected health workforce requirements to provide LTC services to rural dependent elderly in 2020 and 2030

Health workforce	2020 (FTE)	2030 (FTE)
Nurses	14,252	16,094
Doctors	1366	1542
PT	905	1022
CG	44,952	50,148

## Discussion

In Thailand, the LTC system has emphasized the importance of care for older people at home. Home-based health and rehabilitation services for frail elderly are in place. Delivering these services in the home requires a mix of skills amongst those providing the care in the home setting and teamwork between the multidisciplinary health team and CGs.

### Care givers (CG)

Care givers have become an important part of improving accessibility to home health care services in the face of shortages among the mainstream health workforce. CG can work with a multidisciplinary health team to provide integrated social and medical services, as suggested by Fujisawa and Colombo [2]. This approach can help increase accessibility to services for dependent elderly as well as improving patient outcomes.

The implementation of trained CGs in Thailand was unique in the sense that community resources were mobilized to support home health care. VHVs, key players in health system in rural Thailand, were recruited to be CG. Some beneficial effects were identified by CM and CG in using VHV as CG through increased accessibility to care, and CGs have been well accepted by nurses and patients and have a good attitude towards their work. However, CG wages and skill levels were seen as insufficient, and numbers of CGs were perceived to be inadequate.

Importantly, retention of CGs in the home health care workforce in this study was high. Although 11.9% of CGs had left their job in 2018, this figure was lower compared to other countries. A systematic review of health care aides, the primary care givers for dependent older persons, unearthed high turnover rates, ranging from 59.4–100% annually, and there were also high percentages of current health care aides intending to leave their jobs at 33.8%-66.0% [17]. Similarly, in OECD countries, the turnover rate of caregivers was determined to be between 30 and 72% [2]. These figures are repeated in US, with about 71% of nursing assistants leaving work in nursing homes each year [1]. A combination of the working conditions, work-related injuries, low wages, few opportunities for career advancement, and poor work-related benefits contribute to these high rates of health care aides turnover [1, 2, 5, 17]. The high retention rates seen in this study could be influenced by the prior experiences of Thai CGs as VHV, who have previously volunteered to assist with health services in their communities. Their experience with health care service and their good attitude toward the people they serve means that they were well accepted by their clients, which created a good working environment. Moreover, CGs reported that their working time was flexible, and they were able to work close to their hometown. These reasons may contribute to the high retention rates we saw. As the level of remuneration for CGs is much less than for health professionals, having CGs provide home health care can help decrease health system costs while maintaining accessibility to care. Taken together, the results suggest that mobilizing community resources are necessary for an effective LTC system.

### The optimal skill-mix for a multidisciplinary team

The dependent elderly need a range of services, not only assistance with the activities of daily living, but also medical services, sometimes from more than one health care worker. Thus, there is a skill-mix among the various professionals in the multidisciplinary team that is required to meet the needs of each care recipient. This study examined the responsibilities of the home health care team in the delivery of home health care to dependent elderly. We identified the divisions of work between nurses, doctors, PT and CG. Nurses coordinated the multidisciplinary team as a care manager and performed functions ranging from patient assessment, making care plans, case conference coordination, and performing home visits. Doctors and PT were involved in case conferences and home visits. CG played the main role in providing services during home visits. This approach allowed patient treatment to be integrated according to their needs. The service outcomes revealed that most elderly people maintained their ADL levels, and the proportion of those who improved their ADL score was higher than those whose ADL score decreased. The projections for the multidisciplinary health team revealed that in 2030 16,094 nurses, 1542 doctors, 1022 PT and 50,148 CG will be required to look after all of the dependent elderly people in rural Thailand. This represents a challenge for Thailand due to shortages in the health workforce in rural areas, although VHV are mostly available in rural areas.

In situations where countries experience shortages of health care workers, initiatives to optimize the available workforce and achieve the right number and mix of personnel are crucial to providing high-quality care and containing costs [2]. The potential benefits of multidisciplinary teams and, more broadly, of collaboration amongst professionals from different disciplines can be varied, depending on certain contextual conditions [2, 4]. A large systematic review of 14 previous systematic reviews and 33 additional randomized trials by Dubois and Singh [18] that assessed the effectiveness of multidisciplinary teams compared to care provided by a single group of professionals found inconsistent results. Although some systematic reviews found multidisciplinary outpatient teams can improve patient outcomes, this result varied according to the initiatives undertaken and patient conditions. Similarly, a systematic review conducted by Hodgkinson et al. [19] to identify which nursing staffing models (team work among nurses, licensed practice nurses and care givers) were associated with the best patient and staff outcomes found little evidence of the effective use of any of the specific models of care in residential aged care that benefited either residents or care staff.

However, some studies have supported the effectiveness of multidisciplinary teams in relation to aspects such as process outcomes, increased accessibility, and CG acceptability. For instance, a study conducted by Lee et al. [20] evaluated the delegation of work from nurses to trained community care aides in order to support home visits to older persons. The results showed that medicine support visits increased, community care aides were well accepted by nurses and patients, and their visits enabled nurses to devote more time to people with more complex needs. Similarly, a systematic review conducted by Dennis et al. [21] explored the effectiveness of task substitution between doctors and pharmacists and doctors and nurses for the care of older people with chronic diseases. The authors reviewed 46 articles and concluded that task substitution between pharmacists and doctors and nurses and doctors improved the process of care and patient outcomes. According to Fujisawa and Colombo [2], some OECD countries have recognized that a multidisciplinary approach can improve care coordination between social and medical services to the dependent elderly. They recommend that coordination between health care workers, community resources and patients can help to reduce fragmentation of services and to increase communication between providers of LTC. Thus, our results indicate that an approach utilizing multidisciplinary teams together with mobilization of community resources could address some of the challenges of health workforce shortages.

A multidisciplinary health team needs someone to coordinate between the healthcare workers and patients. In our study, nurses acted as CM to fulfill this role. Having a CM to plan, coordinate and manage LTC services has been implemented in several OECD countries. The CM is important for reducing service fragmentation and increasing the communication between care providers and patients in order to ensure that the services provided can best meet the patient’s needs [1, 2].

In relation to health workforce requirements, the results suggest that Thailand should both increase the number of skilled nurses, doctors and PT and improve their distribution in order to provide effective LTC services to the elderly in the near future.

## Conclusion

Increasing demand for aged care and workforce shortages have required a change in thinking about how home health care is organized and delivered to ensure accessibility, quality, and to control costs. Thailand home health care for the dependent elderly living in rural areas has been mobilized as a multidisciplinary health team that delivers comprehensive care. Including paid CGs in the multidisciplinary team crucially contributed to increase accessibility to care. CGs had a good attitude to their work, were well accepted by patients and were likely to remain in the system.

## Data Availability

The data sets used and/or analyzed during the current study are available from the corresponding author on reasonable request.

## Abbreviations

ADL: Activities of daily living
CG: Care giver
CM: Care manager
FTE: Full time equivalent
LTC: Long-term care
PT: Physiotherapist
VHV: Village health volunteer
